# Microglia Gone Awry: Linking Immunometabolism to Neurodegeneration

**DOI:** 10.3389/fncel.2020.00246

**Published:** 2020-08-13

**Authors:** Ruqayya Afridi, Won-Ha Lee, Kyoungho Suk

**Affiliations:** ^1^Department of Pharmacology, Brain Science and Engineering Institute, BK21 Plus KNU Biomedical Convergence Program, School of Medicine, Kyungpook National University, Daegu, South Korea; ^2^School of Life Sciences, BK21 Plus KNU Creative BioResearch Group, Kyungpook National University, Daegu, South Korea

**Keywords:** microglia, phenotype, neurodegeneration, neuroinflammation, metabolism, oxidative stress, homeostatic function

## Abstract

Age-related chronic inflammatory activation of microglia and their dysfunction are observed in many neurodegenerative diseases, and the potential contributions of these dysfunctional cells to neurodegeneration have been demonstrated recently. The housekeeping and defensive functions of microglia, such as surveying the brain parenchyma and phagocytosis of neuronal debris after injury, are important for brain homeostasis and immunity. During neurodegenerative diseases, loss of these functions can promote disease pathology by producing proinflammatory cytokines and increasing oxidative stress, which can exaggerate the ongoing neuroinflammation. A recent surge in microglial research has unraveled myriads of microglial phenotypes associated with aging and neurodegenerative diseases, in addition to the conventional M1/M2 paradigm. Each of these phenotypes can be characterized by distinct transcriptional profiles as well as altered metabolism, migration, and phagocytosis characteristics. Mutations in triggering receptor expressed on myeloid cells 2 (Trem2) and granulin (GRN) are associated with various neurodegenerative diseases, and these genes are dysregulated in the majority of recently identified microglial phenotypes. These genes act as checkpoint regulators and maintain microglial inflammatory fitness, principally through metabolic modulation. Dysfunctional microglia typically show mitochondrial deficits, glycolysis elevation, and lipid droplet accumulation, which results in reduced migration and phagocytosis and increased proinflammatory cytokine secretion and reactive oxygen species release. In this mini-review article, we discuss the existing data regarding metabolic perturbations in dysfunctional microglia and their documented associations with neurodegeneration, highlighting how aging-induced chronic microglial activation alters microglial bioenergetics, leading to impaired homeostatic and housekeeping functions. Dysfunctional microglia initiate or exacerbate neurodegeneration, and key pathways involved in the dysfunctional processes, including metabolism, may represent potential intervention targets for correcting imbalances.

## Introduction

Microglia are brain resident macrophages, comprising 5–12% of all brain cells, that originate from myeloid cells during early development in the yolk sac and possess hematopoietic stem cell (HPSC)-independent self-renewal abilities (Lawson et al., 1990; Sevenich, 2018). Microglia are in constant and dynamic motion, surveying the brain parenchyma through their thin processes (Liu et al., 2019; Bernier et al., 2020). Microglia express several genes encoding various cell receptors, which allow them to sense minute changes in the tissue microenvironment (Haynes et al., 2006; Hickman et al., 2013; Fourgeaud et al., 2016). Homeostatic microglia (HM) serve housekeeping functions and typically express genes involved in synaptic pruning, remodeling, and phagocytosis, including complement component 1q (*C1q*), CX3C chemokine receptor 1 (*Cx3cr1*), and triggering receptor expressed on myeloid cells 2 (*Trem2*), as well as *MER*, *AXL*, β-Hexosaminidase (*HexB*), purinergic receptor P2Y, G protein-coupled 12 (*P2ry12*), S100 calcium-binding protein A8 (*S100A8*), S100 calcium-binding protein A9 (*S100A9*), transmembrane protein 119 (*Tmem119*), G protein-coupled receptor 34 (*Gpr34*), sialic acid-binding Ig-like lectin H (*SiglecH*), and Olfactomedin-like protein 3 (*Olfml3*; Hickman et al., 2013). The homeostatic state of microglia is regulated by certain immune checkpoint regulators that suppress the unnecessary chronic inflammatory activation of microglia. These immune checkpoint regulators maintain microglial activation through direct inhibitory mechanisms mediated by intracellular regulators, including the transcription factor MAF b-ZIP transcription factor B (MafB), microglial/neuronal interactions, including CX3CL1/CX3CR1 and CD200/CD200R interactions, and soluble molecules found in the central nervous system (CNS) milieu [e.g., transforming growth factor-β (TGF-β); Butovsky et al., 2015; Lauro et al., 2015; Matcovitch-Natan et al., 2016; Wang et al., 2020]. Microglial phagocytosis is involved in the elimination of dead cells, damaged axons, and synaptic elements. Diffusible factors released by microglia are associated with neurogenesis, axon fasciculation, synapse maturation, and oligodendrogenesis (Cunningham et al., 2013; Pont-Lezica et al., 2014; Miyamoto et al., 2016; Hagemeyer et al., 2017).

As sentries of the brain, microglial classical inflammatory polarization, occurring in response to tissue insults (tissue damage following trauma and pathogen invasion), may serve as a protective mechanism for brain tissue integrity. The classically activated inflammatory microglia are characterized by enhanced secretion of proinflammatory cytokines, including interleukin-1β (IL-1β) and tumor necrosis factor-α (TNF-α), and increased chemotaxis and phagocytosis, contributing to the clearance of toxic materials following an initial insult. However, this process must be tightly regulated as prolonged and excessive inflammatory activation of microglia can cause host tissue damage, as observed in various neurological disorders (Zhang et al., 2011). “Immune tolerance” is a protective mechanism adapted by the body to reduce immune cell responsiveness, including that of microglia, to subsequent stimulation, limiting tissue damage. In neurodegenerative diseases, chronic neuroinflammatory states are often observed, which are associated with dysfunctional microglial activation. However, the microglial phenotypes identified during aging and neurodegenerative disease appear to differ from the well-known, classic M1/M2 phenotypes. Microglial dysfunction, in terms of reduced chemotaxis and phagocytosis and increased proinflammatory cytokine production, maybe a major contributing factor in various neurodegenerative diseases (Marschallinger et al., 2020).

Immunometabolism has gained much attention, particularly in the context of the phenotypic transitions of innate immune cells, including brain immune cells. The metabolic reprogramming necessary to shift from oxidative phosphorylation to glycolysis during the inflammatory activation of immune cells is a well-established phenomenon. Glycolysis can fuel the energy-intensive processes required for the inflammatory activities of immune cells, including the increased secretion of proinflammatory cytokines and anabolic pathways. In neurodegenerative diseases, hyperglycolytic microglia have been identified by various studies, and these microglia have dysfunctional chemotactic and phagocytotic potentials, highlighting the widespread metabolic perturbations that occur in these tolerant cells, including alterations in oxidative phosphorylation, glycolysis, and the β-oxidation of fatty acids. Thus, the effector functions of microglia are likely to be tightly linked to underlying metabolic pathways. This mini-review article briefly summarizes the current understanding of microglial phenotypes, with a particular focus on microglia associated with neurodegenerative diseases, and the possible roles of altered microglial metabolism during neurodegenerative diseases.

## Microglial Phenotypes Associated With Aging and Neurodegenerative Diseases

### Molecular Features of Microglial Phenotypes: Beyond M1 and M2

Like their peripheral counterparts, macrophages, microglia can adapt to a variety of phenotypes, depending on the cues in the surrounding tissue microenvironment. Each of these phenotypic transitions is associated with a signature gene expression profile, as confirmed by a wealth of recent studies, and plays a variety of roles in each scenario. Traditionally, activated microglia induced by brain damage were classified as either M1 (pro-inflammatory microglia) or M2 (anti-inflammatory microglia). M1 microglia are characterized by the release of proinflammatory cytokines and mediators including reactive oxygen/nitrogen species (ROS/RNS), TNF-α, IL-1β, interleukin-6 (IL-6), and macrophage inflammatory protein 1-α (MIP-1α). The roles of M1 microglia in various neurological disorders have been well-established, and M1 microglia can be beneficial or detrimental, depending on the temporal and regional characteristics of microglia activation. In contrast, the release of IL-10 and IL-4 and the upregulation of cell surface markers, including CD206 and arginase-1 (Arg1), are signature characteristics of M2 microglia. The transition to M2 microglia represents a protective mechanism that neutralizes the inflammatory effects induced by M1 microglia after an insult.

Microglia are highly flexible in terms of utilizing various biomolecules including glucose, lipids, and amino acids for the execution of its effector functions. A recent study demonstrated the shift of microglial metabolism from carbohydrate to amino acid in aglycemic conditions with sustained surveillant function (Bernier et al., 2020). Aging induces gross effects in the brain cellular state, in terms of metabolism and cellular functions. Aging-induced reduced cerebral blood flow and reduced glucose metabolism contribute to the development of neurodegenerative diseases (Camandola and Mattson, 2017). The microglial-primed state of the aging brain further aggravates reduced glucose metabolism because microglia utilize excess glucose to maintain the inflammatory state (Kleinberger et al., 2017). Recent reports indicate that during aging and age-related neurodegenerative diseases, microglia can change from HM to other reactive states, including activated response microglia (ARM), disease-associated microglia (DAM), the microglial neurodegenerative type (MGnD), lipid-accumulating microglia (LDAM) and dark microglia (Keren-Shaul et al., 2017; Krasemann et al., 2017; Deczkowska et al., 2018; Sala Frigerio et al., 2019; Marschallinger et al., 2020), which can be characterized based on differential transcriptomic profiles, morphology, and functions. The dysfunctional microglial phenotypes observed in neurodegenerative diseases are induced by the phagocytosis of apoptotic neurons. The molecular signatures associated with dysfunctional microglia resemble microglial gene expression profiles observed during development, with increased apolipoprotein E (*ApoE*) levels and reduced homeostatic gene expression (Butovsky et al., 2014). APOE induction and TGF-β suppression have been identified as upstream regulators of MGnD, as revealed by the transcriptional profiling of microglial cells. The expression of *Olfml3*, *P2ry12*, *Tmem119*, myocyte enhancer factor 2A (*Mef2a*), and spalt like transcription factor 1 (*Sall1*), which are TGF-β-dependent genes, decreased in MGnD, whereas the expression of APOE-dependent genes, including C-type lectin domain family 7 (*Clec7a*), and *Axl* increased. The signature genes associated with MGnD regulate lipid metabolism and phagocytosis. Another microglial phenotype in the aging brain has been recently identified, referred to as lipid-droplet-accumulating microglia (LDAM), which differ from both DAM and MGnD in terms of transcriptional profile but show similar characteristics of reduced phagocytosis and altered lipid metabolism, indicating a correlation between the underlying metabolic pathways and microglial effector functions (Wang et al., 2015). The dark microglia are named after their dark appearance under an electron microscope (Bisht et al., 2016; El Hajj et al., 2019). The dark microglia are found abundantly in aged and AD mice with down-regulated homeostatic genes including CX3CR1, and P2RY12 (Paolicelli et al., 2014; Stratoulias et al., 2019). Because the studies that identified microglial phenotypes primarily employed aging animals or animal models of AD, future studies may unravel the molecular signatures of additional microglial phenotypes, associated with other neurodegenerative diseases, as dysfunctional microglia are evident in all neurodegenerative diseases.

### Impaired Migration and Phagocytosis in Dysfunctional Microglia: The Role of Metabolism

**Figure 1:**
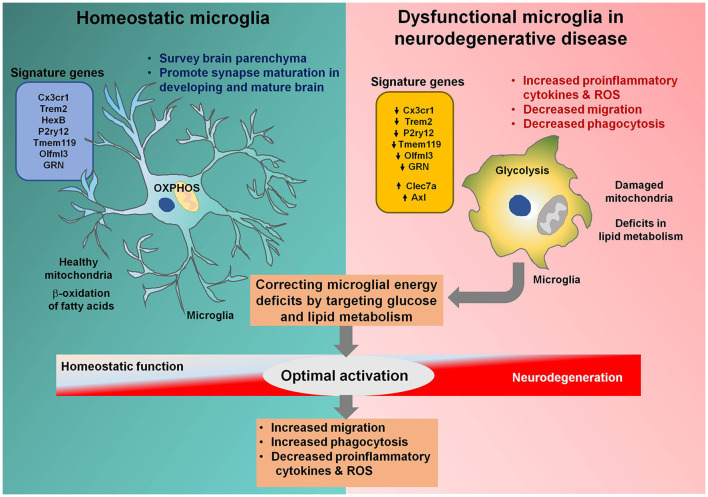
Microglial metabolic modulation as a strategy for the optimal activation of microglia in neurodegenerative diseases. Homeostatic microglia (HM) maintain brain homeostasis by surveying the brain parenchyma. HM rely on oxidative phosphorylation and healthy mitochondria to efficiently perform the β-oxidation of fatty acids. Microglia become activated during neurodegenerative diseases, resulting in increased glycolysis and mitochondrial damage. Triggering receptor expressed on myeloid cells 2 (*Trem2*) and granulin (*GRN*) are downregulated in dysfunctional microglia associated with neurodegenerative diseases, halting their transition to the optimal activation state. Dysfunctional microglia can no longer sustain their energy requirements and lose migratory and phagocytotic activities. Correcting energy deficits in microglia can help microglia achieve the optimal activation state, with reduced proinflammatory cytokine and reactive oxygen species (ROS) release and increased phagocytotic and migratory potential, alleviating neurodegenerative diseases.

Misfolded protein aggregation is a common feature of various neurodegenerative diseases and is associated with the activation of microglial innate immune receptors, including Toll-like receptors (TLR) 2 and 4 (Yerbury et al., 2016). The role of microglial activation in various neurodegenerative diseases remains elusive, and many studies have identified beneficial roles of microglial activation during early neurodegeneration (Keren-Shaul et al., 2017). The clustering of microglia around protein aggregates, followed by phagocytosis, may reduce the accumulation of misfolded proteins during neurodegenerative diseases, implying the importance of microglial migration and phagocytosis for age-related neurodegenerative diseases. Increased microglial migration and phagocytosis are both bio-energetically expensive processes; therefore, microglial metabolic perturbations during neurodegenerative diseases are not surprising (Figure 1). Common metabolic features observed for microglia associated with disease models of AD, Huntington’s disease (HD), and amyotrophic lateral sclerosis (ALS) include compromised mitochondrial bioenergetic pathways and enhanced glycolysis (Joshi et al., 2019). Enhanced mitochondrial fragmentation was recently identified in cellular and animal models of neurodegenerative diseases, associated with the increased proinflammatory cytokine production, resulting in increased neurotoxicity (Joshi et al., 2019). The mitochondrial perturbations observed in activated microglia can propagate signals to other cell types, including astrocytes and neurons, further exaggerating the disease outcome (Joshi et al., 2019). Increased glycolysis and upregulated expression of glucose transporters as well as glycolytic enzymes in activated microglia are a well-established phenomenon (Afridi et al., 2020). Increased microglial glycolysis is often coupled with the increased secretion of proinflammatory cytokines, exacerbating neurotoxicity, and escalating ongoing neurodegeneration. Microglial migration and phagocytosis demand more than the normal amounts of energy, although whether glycolysis or oxidative phosphorylation promotes these processes remains unclear. Glycolysis was increased in microglial cells during the early phase of AD, coupled with increased phagocytic activity followed by an “immune tolerant” phase, during which glycolysis and oxidative phosphorylation were both disturbed, with less phagocytic potential (Baik et al., 2019). Microglial functions were restored through increasing metabolic functions with interferon-gamma (IFN-γ), resulting in increased microglial clustering around AD plaques, phagocytosis, and TNF-α production. Although the phagocytic potential of microglia was restored through metabolic boosting, the effects of the resultant increase in proinflammatory cytokine release remained unclear. Conversely, in AD models, oxidative phosphorylation increased phagocytosis and migration but reduced proinflammatory cytokine production (Pan et al., 2019). These contradictory results indicate that although metabolic reprogramming may be a tool that can be used to treat various pathologies involving microglial cells, the identification of the metabolic pathways remains necessary to determine which pathways are required for optimal microglial activation states, resulting in intact migratory and phagocytic properties and reduced proinflammatory cytokine production.

Mutations in genes that regulate microglial homeostatic functions have been associated with aging-related neurodegenerative diseases. *Trem2* and granulin (*GRN*) are responsible for the maintenance of microglia in the homeostatic state or the conversion to DAM, depending on the tissue microenvironment and the instigating stimuli (Lui et al., 2016; Ulland et al., 2017). *Trem2* regulates important microglial functions, including migration, survival, chemotaxis, synaptic pruning, and proliferation (Kleinberger et al., 2014, 2017; Mazaheri et al., 2017; Filipello et al., 2018). Trem2 is an innate immune receptor and cell surface protein, which acts through its co-receptor DNAX activation protein of 12 kDa (DAP12; Yeh et al., 2016). *Trem2* is an important gene, involved in the regulation of the microglial transition to DAM, which is characterized by increased migration and phagocytosis, *via* the regulation of a distinct set of genes (Deczkowska et al., 2018; Ulland and Colonna, 2018). In the animal model of AD, *Trem2*-deficient microglia were unable to transition to a late-stage DAM profile (DAM stage 2), as confirmed by single-cell RNA sequencing (scRNA-seq) analysis (Keren-Shaul et al., 2017). *Trem2*-deficient microglia remained in an intermediate DAM state (DAM stage 1; Keren-Shaul et al., 2017), which represents the initial activation of microglia and is independent of *Trem2*, characterized by the increased expression of transmembrane immune signaling adaptor (*Tyrobp*) and *Apoe* and the downregulation of *Cx3cr1* (immune checkpoint genes; Keren-Shaul et al., 2017). *Trem2* regulates the full activation of the DAM (DAM stage 2) by upregulating the gene expression necessary for phagocytosis and lipid metabolism (Keren-Shaul et al., 2017). Moreover, *Trem2* absence in the developing brain results in impaired synaptic elimination with enhanced excitatory neurotransmission (Filipello et al., 2018). Progranulin (PGRN) is a growth factor-like protein with neurotrophic functions that regulate lysosomal proteins in the brain (Van Damme et al., 2008; Hu et al., 2010; Zhou et al., 2015). PGRN is proteolytically processed into GRN. Mutations in *GRN* have been associated with various neurodegenerative and neuroinflammatory disorders, including AD, ALS, Creutzfeldt–Jakob disease, and viral infections of CNS (Johnston et al., 2001; Malaspina et al., 2001; Baker and Manuelidis, 2003; Ahmed et al., 2007).

*Trem2* maintains microglial immune fitness, enhancing the ability to engulf misfolded protein aggregates through the modulation of metabolic machinery in neurodegenerative diseases. The activation of *Trem2* is induced by molecules with lipid backbones, produced as a result of neuronal and glial damage (Wang et al., 2015). *Trem2* has recently gained much attention for its role in the regulation of microglial metabolic fitness in AD and the aging brain. *Trem2* variants have been associated with dysfunctional microglia, leading to early neurodegeneration in AD. *Trem2* fulfills microglial ATP requirements in AD by upregulating mammalian target of rapamycin (mTOR) signaling through upstream activators, such the serine/threonine-protein kinase B (Akt), pyruvate dehydrogenase kinase-1 (PDK1), and phosphatidylinositol 3-kinase (PI3K), which are recruited by the Trem2-associated signaling subunits DAP10 and DAP12 (Peng et al., 2010). Microglia isolated from *Trem2*-deficient AD mice showed extensive dysregulation among genes that regulate glucose metabolism, including hexokinase-1, pyruvate kinase M2, and lactate dehydrogenase A (Ulland et al., 2017). *Trem2* maintains microglial survival and activation, which is challenged during neurodegenerative diseases. *Trem2*-deficient microglia showed the increased cholesterol accumulation, in the form of cholesteryl ester (CE), compared with wild-type microglia when challenged with myelin, *in* *vitro* (Nugent et al., 2020). In addition to heightened CE accumulation, components of other important pathways that regulate lipid transport and metabolism, including *Apoe*, apolipoprotein C1 (*Apoc1*), cholesterol 25-hydroxylase (*Ch25h*), lipase A (*Lipa*), neutral cholesterol ester hydrolase 1 (*Nceh1*), Niemann-Pick Disease Type C2 (*Npc2*), and sterol O-acyltransferase 1 (*Soat1*), were downregulated in *Trem2*-deficient microglia (Nugent et al., 2020). The reduced metabolic flux of CE may expose it to oxidation, resulting in the secondary accumulation of oxCE species, as observed in the *Trem2*^−/−^ mouse brain (Nugent et al., 2020).

*GRN* is involved in the regulation of microglial homeostatic functions (Pickford et al., 2011; Götzl et al., 2019). Microglia isolated from *Grn*^−/−^ mice exhibit hyperactivation state associated with the downregulation of the homeostatic *P2ry12* gene (Götzl et al., 2019). Signature genes expressed in MGnD were upregulated in *GRN*-deficient microglia, including *Apoe*, *Clec7a*, and *Trem2* (Götzl et al., 2019). *GRN*-deficient microglia demonstrated increased migration, phagocytosis, and clustering around amyloid β (Aβ) plaques (Götzl et al., 2019).

Dysfunctional microglia were recently identified in aging mice, with increased lipid droplet accumulation, named LDAMs (Marschallinger et al., 2020). LDAMs produced increased levels of proinflammatory cytokines and ROS, similar to LPS-treated microglia, indicating the primed state of microglia during aging. Moreover, several genes regulate the LDAM phenotype, including *GRN*, solute carrier family 33 Member 1 (*SLC33A*), sorting nexin 17 (*SNX17*), vacuolar protein sorting retromer complex component (*VPS35*), *NPC2*, and ceroid lipofuscinosis neuronal 3 (*CLN3*), with some genes overlapping between DAM and MGnD. The pathways regulating carbohydrate metabolism and fatty acid oxidation were disturbed in microglia derived from *Grn*^−/−^ mice, further increasing the likelihood that *GRN* acts as a regulator of microglial metabolic function. Dysfunctional effector functions of microglia, including impaired phagocytosis, were also identified in LDAMs, indicating the pivotal roles of age-associated microglial phenotypes during neurodegeneration.

### Dysfunctional Microglia in Neurodegenerative Diseases

The microglial contribution to various neurodegenerative diseases, including AD and demyelinating diseases, is evident. Microglial inflammatory activation leads to increased proinflammatory cytokine production, exacerbating the ongoing neurodegeneration. Recent studies have highlighted dysfunctional microglia, with decreased chemotactic and phagocytotic activities, resulting in increased Aβ deposition in the AD brain. The loss-of-function of microglia-specific genes, including *Trem2* and *GRN*, is associated with excessive neurodegeneration in AD. The increased autophagosome accumulation in *Trem2*-deficient microglia implies metabolic stress, which is further supported by the decreased expression of genes involved in energy metabolism and biosynthesis (Keren-Shaul et al., 2017). Correcting ATP deficits in *Trem2*-deficient microglial cells through cyclocreatine treatments restored the microglial response to Aβ plaques in AD mice, indicating that metabolic deficits are the primary cause of dysfunctional microglial responses regulated by *Trem2* in AD (Keren-Shaul et al., 2017). Microglial *GRN* loss-of-function causes hyperinflammatory activation in aging mice, leading to neurotoxicity, which highlights the plausible roles of *GRN* during microglial phenotypic transitions (Götzl et al., 2019). Loss of microglial *GRN* induces widespread disturbances in energetic pathways, leading to lipid droplet accumulation in microglia (Marschallinger et al., 2020). In addition to AD pathology, dysfunctional microglia have been implicated in other neurodegenerative diseases, including frontotemporal dementia (FTD). In *Grn*^−/−^ mice, an FTD model, dysfunctional and pro-inflammatory microglia were identified demonstrating increased lipid droplet accumulation (Marschallinger et al., 2020). These microglial cells demonstrated defective phagocytosis and produced high levels of ROS and proinflammatory cytokines (Marschallinger et al., 2020). Targeting microglial responses through metabolic reprogramming may be a promising therapeutic intervention for AD and other neurodegenerative diseases; however, intensive research remains necessary to elucidate the precise links between metabolic perturbations and effector functions in microglia.

## Conclusions and Future Perspectives

Recent genetic findings highlight the crucial roles of microglia in neurodegenerative diseases. Genetic mutations in *Trem2* and *Grn* result in dysfunctional microglia, with altered metabolism and the increased secretion of proinflammatory cytokines and ROS. Various microglial activation states associated with aging and neurodegenerative diseases have been reported recently, with similar or overlapping transcriptomic profiles, including the dysregulation of genes regulating metabolism and phagocytosis. However, research gaps remain in the unified description of microglia associated with neurodegeneration, as well as in the exact mechanisms that link metabolism to microglial effector functions. Based on recent studies, both the “over-activation” and the “over-silencing” of microglia will not be effective in the fight against neurodegenerative diseases. The heterogenous microglial phenotypes identified in various disease states in recent studies require future research to reveal key regulators of these phenotypes which can be easily targeted. The optimal microglial activation state, which exerts beneficial effects against various disease anomalies, remains to be identified, necessitating future studies that may unravel microglial metabolic pathways.

## Author Contributions

All authors listed have made a substantial, direct and intellectual contribution to the work, and approved it for publication. RA, W-HL, and KS formulated the focus of this review. RA and KS conducted the literature review and participated in the discussion. RA and KS wrote the manuscript.

## Conflict of Interest

The authors declare that the research was conducted in the absence of any commercial or financial relationships that could be construed as a potential conflict of interest.
